# The energy transfer mechanism of a photoexcited and electroluminescent organic hybrid thin film of blue, green, and red laser dyes

**DOI:** 10.1186/s11671-015-0899-y

**Published:** 2015-04-23

**Authors:** Weiling Li, Jing Zhang, Yanqiong Zheng, Guo Chen, Miao Cai, Bin Wei

**Affiliations:** School of Materials Science and Engineering, Shanghai University, 149 Yanchang Road, Shanghai, 200072 People’s Republic of China; Key Laboratory of Advanced Display and System Applications, Ministry of Education, Shanghai University, 149 Yanchang Road, Shanghai, 200072 People’s Republic of China; School of Mechanical and Electrical Engineering, Guilin University of Electronic Technology, 1 Jinji Road, Guilin, 541004 People’s Republic of China

**Keywords:** Laser dyes, Cascade energy transfer, Hybrid thin films, OLEDs

## Abstract

Though optically pumped lasing has been realized for years, electrically pumped lasing has not yet been achieved in organic semiconductor devices. In order to make a better understanding of the laser mechanisms of the organic materials, we prepared organic thin films consisting of three efficient laser dyes of a blue emitter, 4″,4″′-N,N-diphenylamine-4,4′-diphenyl-1,1′-binaphthyl (BN), a green emitter, 1,4-bis[2-[4-[N,N-di(p-tolyl)amino] phenyl]vinyl]benzene (DSB), and a red emitter, 4-(dicyanomethylene)-2-t-butyl-6(1,1,7,7-tetramethyljulolidy-l-9-enyl)-4H-pyran (DCJTB) with different doping concentrations for the first time to investigate the cascade energy transfer process. The energy transfer schemes in the co-doped thin films in photoluminescence and electroluminescence have been investigated. The results indicated that the DSB molecules acted as a bridge to deliver energy more effectively from the host (BN) to the guest (DCJTB). Meanwhile, the maximum current efficiency (*C*_E_) and power efficiency (*P*_E_) of the organic light-emitting devices (OLEDs) with the emitting layer of lower doping concentration were 13.5 cd/A and 14.1 lm/W, respectively.

## Background

The realization of electrically pumped lasing is still considered as a significant challenging research subject in the field of organic semiconductor devices, though various approaches have been attempted [1-3]. At present, stimulated emission behavior, including amplified spontaneous emission (ASE), is observed in organic lasing dyes [4-10], yet the stimulated emission is eventually hampered by the low-efficiency energy transfer mechanism and concomitant singlet-triplet annihilation, which prohibits a sustained population inversion [11,12]. In addition, the low efficiency problem caused by annihilation can be resolved by adding an auxiliary film-forming polymer to dilute the laser dye mixed solutions [13,14]. On the other hand, from the perspective of organic materials, the less overlap between absorption spectra and photoluminescence (PL) spectra will help reduce the self-absorption attenuation of this material, ensuring the stimulated lasing with low threshold. Therefore, the study on the optical as well as electrical characteristics of organic dyes would be crucial for the realization of electrically driven organic lasers [15-18].

Here, we demonstrated a cascade host-guest energy system by mixing three high-efficiency organic laser dyes (4″,4″′-N,N-diphenylamine-4,4′-diphenyl-1,1′-binaphthyl (BN), 1,4-bis[2-[4-[N,N-di(p-tolyl)amino] phenyl]vinyl]benzene (DSB), and 4-(dicyanomethylene)-2-t-butyl-6(1,1,7,7-tetramethyljulolidy-l-9-enyl)-4H-pyran (DCJTB)) in an organic polymer film. Firstly, we investigated the optical properties of the neat thin films of BN, DSB, and DCJTB, including absorbance and PL spectra. Energy transfer is confirmed by the large overlap between the PL spectrum of BN and the absorption spectra of DSB and DCJTB. Then, we fabricated hybrid-conjugated polymer films by doping DSB and DCJTB into BN, and their optical characteristics were obtained. Moreover, the cascade energy transfer mechanism in these co-doped films was studied. In addition, we inserted the hybrid thin film into organic light-emitting devices (OLEDs) serving as the light-emitting layer to further explore the effect of cascade energy transfer in the co-doped system. The results show that the DSB molecule acts as both an interstitial molecule and an energy delivery medium, playing an important role in the cascade Förster energy transfer system. The electroluminescence (EL) spectra exhibit no BN’s characteristic peaks, but mainly DCJTB’s. It is considered that the light energy of DCJTB and DSB in EL is not only from energy transfer among dyes but also from carrier trap recombination.

## Methods

Prepared glass substrates were cleaned by detergent, de-ionized water, acetone, and isopropanol, and then dried in a drying cabinet. Before loading into a custom-made high-vacuum thermal evaporation chamber, the substrates coated with indium-tin-oxide (ITO, 15 Ω/□, 150 nm) were treated in a UV-ozone environment for 15 min. The OLEDs share the structure of ITO/2T-NATA (20 nm)/NPB (30 nm)/emitting layer (20 nm)/TPBi (30 nm)/LiF (0.3 nm)/Al (150 nm). In the OLEDs, 4,4′,4″-tris(N-(2-naphthyl)-N-(phenylamino)-triphenyl-amine) (2T-NATA), N,N′-diphenyl-N,N′-bis(1-naphthyl)-1,1′-biphenyl-4, and 4″-diamine(NPB),1,3,5-tris(2-N-phenylbenzimidazolyl) benzene (TPBi) act as a hole injection layer [19], a hole transportation layer, and an electron transportation layer, separately. A thin layer of 0.3 nm of LiF is inserted between the active layer and cathode to form an ohmic contact. The OLEDs were fabricated by conventional vacuum deposition under a base pressure lower than 1.0 × 10^−5^ mbar. The typical deposition rates of organic materials, lithium (LiF), and Al were 0.6, 0.1, and 5 Å/s, respectively.

The absorption and PL spectra were measured by a HITACHI F-4500 fluorescence spectrophotometer (Hitachi, Ltd., Tokyo, Japan). The active area of the OLEDs defined by the overlap between the cathode and the anode was 4 mm^2^. The current density-voltage-luminance (*J*-*V*-*L*) and current efficiency-luminance-power efficiency (*C*_E_-*L*-*P*_E_) were measured by a testing setup consisting of a Keithley 2400 SourceMeter (Keithley Instruments, Inc., Cleveland, OH, USA) and a Photo Research PR-650 spectrophotometer (Photo Research, Inc., Chatsworth, CA, USA). All the measurements were performed in air at a room temperature of 25°C without device encapsulation.

## Results and discussion

**Figure 1:**
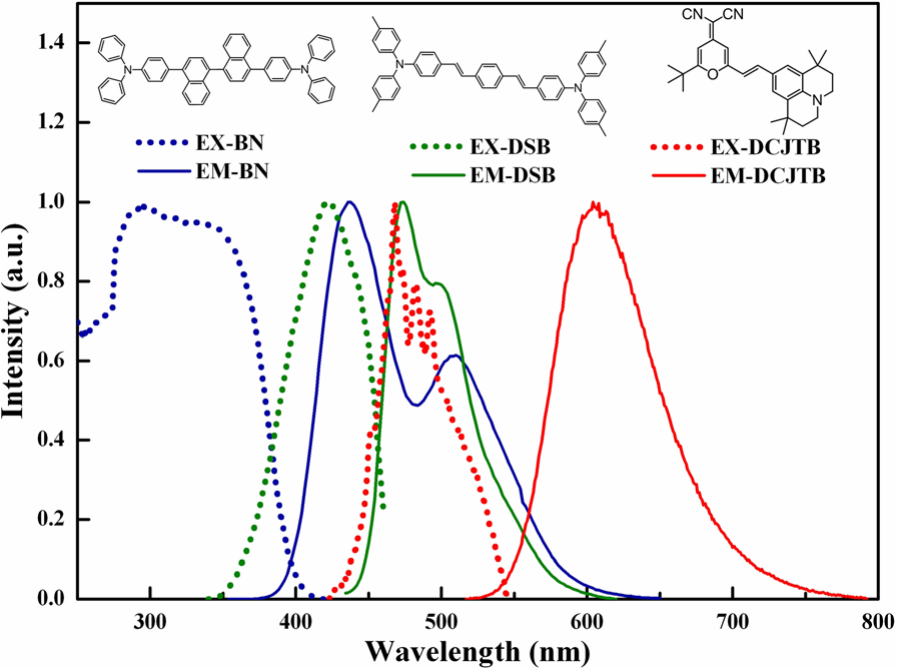
Absorbance (dotted line) and PL (solid line) spectra of the neat BN (blue), DSB (green), and DCJTB (red) films.

For an efficient Förster energy transfer from the host to the dopant, there should be a strong overlap between the PL spectrum of the host and the absorption spectrum of the dopant [20,21], which is confirmed in Figure 1. BN has a strong emission in the deep blue (436 nm) region, followed by a shoulder at 510 nm, while the absorption peak of DCJTB is fitly at 468 nm, and the spectra overlap ratio (the overlap area of the spectra between the absorption of the guest and the emission of the host divides the area of the emission spectra the host) is 86.05%, providing possibilities for efficient energy transfer.

**Figure 2:**
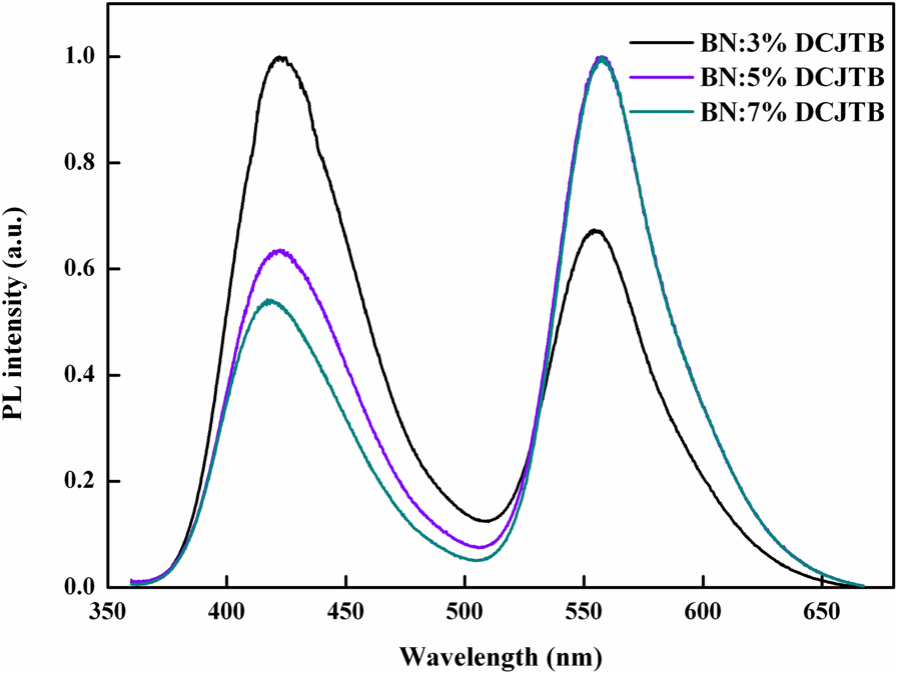
PL spectra of the DCJTB-doped BN thin films with different concentrations.

To verify this assumption, the DCJTB-doped BN thin films with different doping concentrations are pumped at the excitation wavelength of BN (*λ*_BN_). As shown in Figure 1, the absorption coefficient of DCJTB at *λ*_BN_ is near to 0, so DCJTB is not supposed to be excited. However, as shown in Figure 2, there is an impressive characteristic peak at 570 nm. Therefore, the emergence of the emission peak at 570 nm can be seen as a characteristic signature of DCJTB resulting from energy transfer. Besides, along with the increasing of concentration, the red emission from the DCJTB enhances. We discover that the overlap between the PL spectrum of BN and the absorption spectrum of DSB is large and the superposition between the PL spectrum of DSB and the absorption spectrum of DCJTB is enormous as well, with the spectra overlap ratios of 61.68% and 91.92%, respectively. These results inspire us to further study the cascade energy transfer schemes by introducing DSB as a bridge in the energy transfer system.

**Figure 3:**
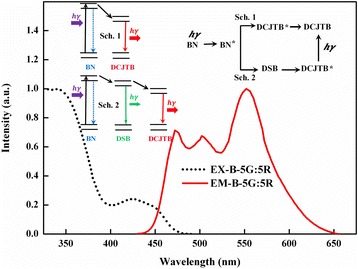
PL spectra of the DSB- and DCJTB-doped BN thin films with a concentration of 100:5:5; the insets are the description for the two schemes of energy transfer system.

Figure 3 demonstrates the cascade energy transfer by using a three-lasing-dye-co-doped thin film excited with *λ*_BN_. An excitation peak can be clearly observed, consistent with the one in neat BN films, and the emission peaks at 470, 520, and 570 nm, which is in accordance with the emission peaks of DSB and DCJTB. These are evidences for the efficient energy transfer in co-doped films, since the emission spectrum characteristics of BN vanish.

We consider that there are two schemes in this system facilitating efficient energy transfer and define them as schemes 1 and 2, respectively, as shown in the insets of Figure 3. DCJTB receives energy from BN directly, while DSB allows Förster energy transfer from BN and also acts as a bridge to deliver energy to DCJTB, resulting in the stronger emission of DCJTB than that of DSB. In experiments, when introducing the bridge DSB material, the concentrations of the other two dyes are decreased correspondingly, preventing the concentration quenching of the emission. The cascade energy transfer mechanism separates the emission spectra from the absorption spectra; thus, the self-absorption loss of materials can be avoided. It deserves to be mentioned that the peak of the PL spectra for DCJTB has a blue shift, which has affinity with the high concentration of lasing dyes. Once the energy bridge DSB cannot pass on the energy sufficiently, green emission appears, resulting in the blue shift.

**Figure 4:**
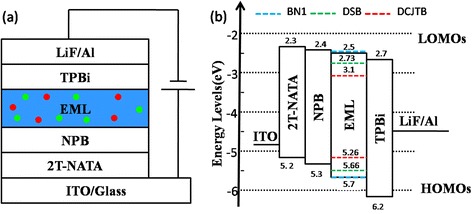
Device architecture and energy level diagram. **(a)** Architecture of the OLEDs is ITO/2T-NATA (20 nm)/NPB (30 nm)/emitting layer (20 nm)/TPBi (30 nm)/LiF (0.3 nm)/Al (150 nm), and the doping concentrations of the emitting layer (EML) of BN:DSB:DCJTB are 100:3:0.21 for device A and 100:20:4 for device B. **(b)** Device energy level diagram.

Researchers have revealed that employing laser dyes in organic semiconductor devices as the light-emitting layer can improve the performances of OLEDs. Here, we fabricated two OLEDs to explore the electroluminescent characteristics of the co-doped thin films. The ratios of the doping concentrations of BN:DSB:DCJTB in EML are 100:3:0.21 for device A and 100:20:4 for device B, respectively. The architecture and the energy level diagram of the devices shown in Figure 4 indicate the excellent energy matching between the materials, thus promoting carrier injection and transportation [22].

**Figure 5:**
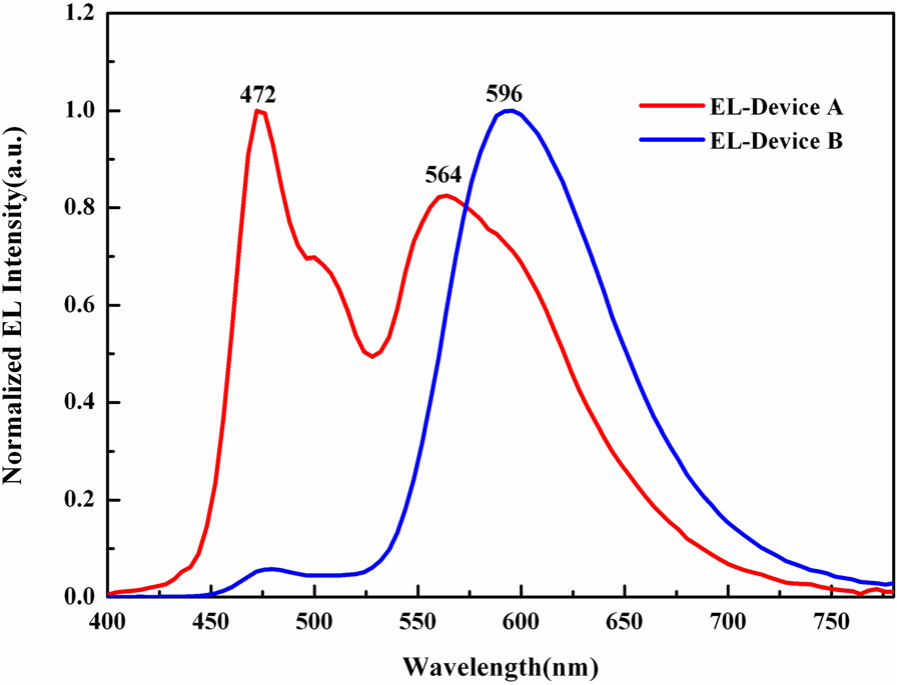
Electroluminescence spectra of devices A and B under the same voltage.

Figure 5 shows the EL spectra of devices A and B under the same voltage. The emission peak of DCJTB can be clearly seen in both spectra, which indicates efficient energy transfer among the laser dyes. But by comparing the EL spectra with the PL spectra, it is found that the emission peaks of device A are consistent with those in the PL spectra. However, when raising the concentrations of DSB and DCJTB (device B), the emission of BN and DSB is greatly decreased, resulting in the strong emission of DCJTB. Therefore, we conclude two energy transfer mechanisms in EL: a) DSB and DCJTB can directly trap carriers to emit and b) when BN is excited, the energy of BN stimulates DSB and DCJTB to emit green and red light; in the meantime, DCJTB is used as a carrier trap to attract the hole from BN, resulting in greater probabilities of DCJTB’s emission and improving the energy efficiency of BN. It also can be observed that the emission peak of DCJTB is at 596 nm in device B, corresponding to a red shift of 32 nm to device A, which peaks at 564 nm. Due to the non-planar structural characteristics of the DCJTB molecule, increasing the concentration more likely leads to molecular aggregation and exciplex formation, making the band gap of DCJTB smaller. From the perspective of energy transition, BN and DSB deliver energy to DCJTB more sufficiently in OLEDs with a higher doping concentration of DCJTB (device B), thus raising the possibilities of emission of monochromatic light.

**Figure 6:**
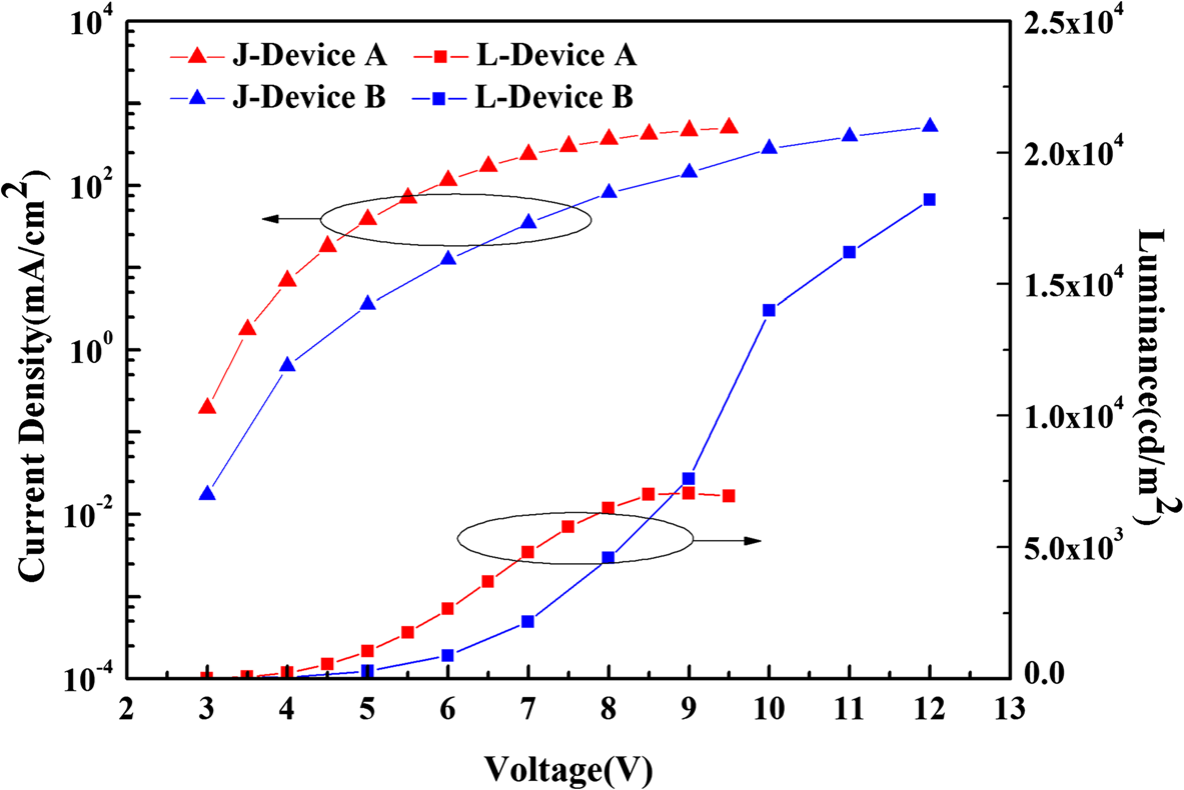
The *J-V-L* curves of devices A (red line) and B (blue line).

**Figure 7:**
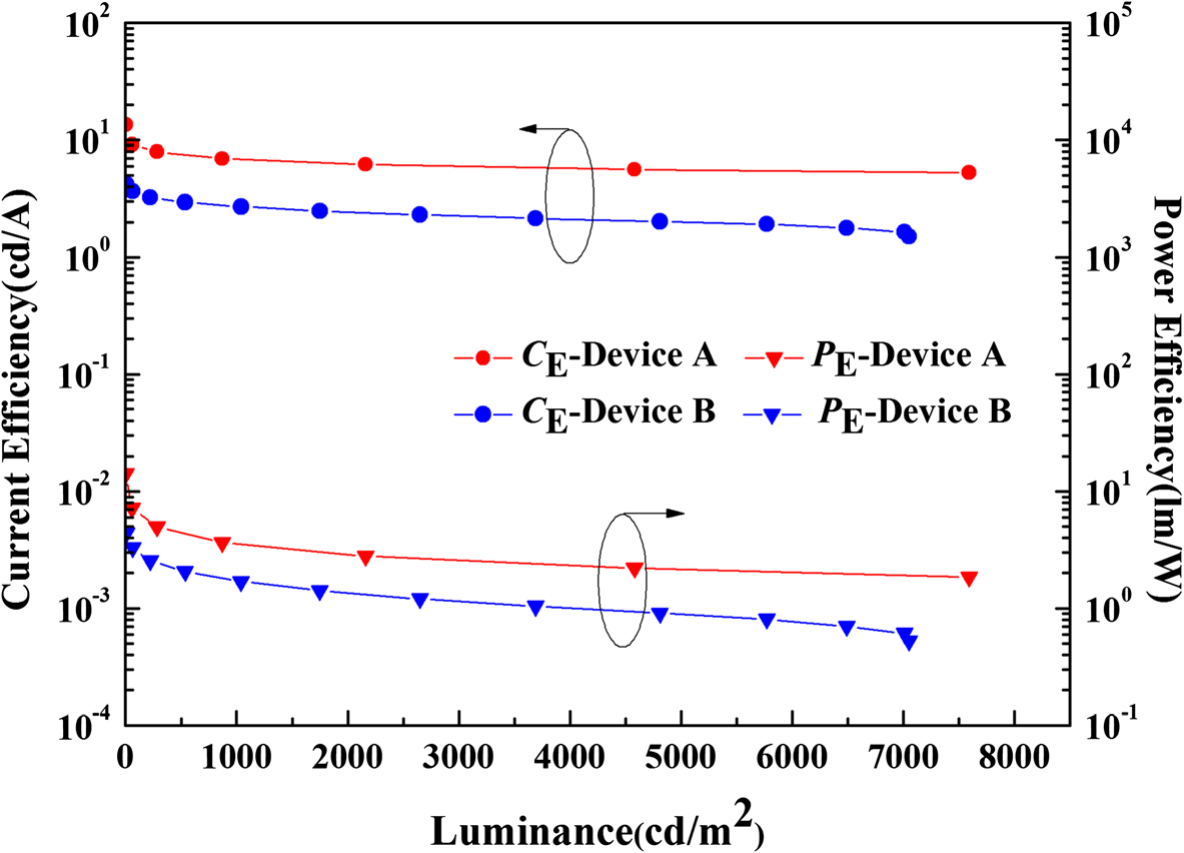
The *C*_E_-*L*-*P*_E_ curves of devices A (red line) and B (blue line).

Figures 6 and 7 reveal the *J*-*V*-*L* and *C*_E_-*L*-*P*_E_ properties to further study the influences of doping concentrations on OLEDs. Applying the same voltage, device B shows lower current density than device A. Figure 6 manifests that device B can suffer from a higher voltage and it obtains the maximum of 18,700 cd/cm^2^ at 12 V. Device B possesses a higher doping concentration of DCJTB of which the band gap is only 2.1 eV. A low band gap implies that DCJTB molecules are more prone to electronic transitions to form excitons than DSB and BN.

With the increasing doping concentration of DSB and DCJTB molecules, luminance is significantly improved, while *P*_E_ and *C*_E_ are decreased, as shown in Figure 7. Hence, both the *C*_E_ and *P*_E_ of device A are higher than those of device B. Maximum values of *C*_E_ and *P*_E_ of device A are 13.5 cd/A and 14.1 lm/W, while 4.2 cd/A and 4.4 lm/W are achieved for device B. Obviously, both of the OLEDs present a steady performance without roll-off phenomenon, which is essential for the further optimization of the device performances.

Therefore, the larger concentration of DCJTB results in a stronger emission, while less amount helps enhance the stabilities and the efficiencies of the devices. This requires us to make a trade-off to obtain better luminescent properties.

## Conclusions

We have developed a novel cascade energy transfer system by mixing three high-efficiency organic lasing dyes. Through investigating the DSB- and DCJTB-doped BN thin films with different doping concentrations, we have demonstrated that there were two kinds of energy transfer schemes in the co-doped thin films in PL: a) DCJTB receives energy from BN directly and b) DSB allows Förster energy transfer from BN and delivers energy to DCJTB. Compared with the PL characteristics of the DCJTB-doped BN thin films, a more efficient energy transfer mechanism was manifested in the co-doped system with the bridge DSB molecules. Meanwhile, this co-doped system separated the emission spectra of the guest from the absorption one, which decreased the self-absorption attenuation of materials to further lower self-absorption. In addition, the co-doped OLEDs were fabricated. Two schemes of energy transfer were concluded in EL: a) DSB and DCJTB can directly trap carriers to emit light and b) when BN is excited, excitons of BN stimulate DSB and DCJTB to emit green and red light, and DCJTB is used as a carrier trap to attract the hole from BN, resulting in greater probabilities of DCJTB’s emission, as well as the improvements of the energy efficiency of BN. Both the trap recombination and energy transfer mechanisms work in EL, while the energy transfer dominates in PL. A peak current efficiency of 13.5 cd/A and a power efficiency of 14.1 lm/W were obtained in lower doping concentrations of device A. However, in higher doping concentrations of device B, the OLEDs showed a stronger emission. It implies that adjusting the mass ratio of the laser dyes can improve the performances of the hybrid thin film in OLEDs or organic lasing, and we will then study the laser characteristics of the co-doped thin films.

## Abbreviations

ASE: amplified spontaneous emission
*C*_E_*-L-P*_E_: current efficiency-luminance-power efficiency
EL: electroluminescence
*J*-*V*-*L*: current density-voltage-luminance
OLEDs: organic light-emitting devices
PL: photoluminescence
